# 379. Abstract For Comparison of Mandatory vs Non-Mandatory Compliance Rates For SARS-CoV-2 Testing in Grades K-12

**DOI:** 10.1093/ofid/ofab466.580

**Published:** 2021-12-04

**Authors:** Jennifer Veltman, Philip Papayanis, Alex Dubov

**Affiliations:** 1 Wayne State University School of Medicine, Detroit, Michigan; 2 Loma Linda University School of Medicine, Loma Linda, California; 3 Loma Linda University, Loma Linda, California

## Abstract

**Background:**

Rapid testing to identify asymptomatically infected students with SARS-CoV-2 in elementary schools has been suggested as a possible method to reduce risk for in person instruction. As of August 3, 2020 (updated on January 25, 2021), California schools who obtained a waiver to conduct in-person instruction are not required to have mandatory testing for asymptomatic students, except for high contact sports which are required to undergo weekly testing. We explored the uptake of voluntary vs mandatory testing in a private waivered school.

**Methods:**

Between the dates January 25, 2021 to April 16, 2021, the K-12 school superintendent sent an email to all parents outlining the voluntary testing program with a link to the on-line sign up and consent form. All students were offered weekly self-collected anterior nares BinaxNOW Rapid Antigen Test. Signed parental consent was required and tests were performed at the school. Students participating in contact sports were required to undergo testing the week a varsity game was played as a condition of participation. Data was gathered from the school administration and de-identified.

**Results:**

K-5 Lower school had a school population of 448 students. Testing was offered on 8 weeks during the period of 2/15-2/19 to 4/5-4/9. 2 students (0.45%) receive screening on the week of 3/22-3/26. The other seven weeks when screening was offered 0 students received screening. 6-12 Upper school had a school population of 360 enrolled students. Testing was offered 3/8-3/12 and 3/15-3/19. The upper school had 22 students (6.11%) receive testing on the week of 3/8-3/12 and 21 students (5.83%) on the week of 3/15-3/19. Contact sports teams had 67 students on their roster. Weekly testing was offered from 3/22-3/26 to 4/12-4/16. Contact sports teams had 10 students (14.93%) receive testing on the week of 3/22-3/26, 33 students (52.24%) on the week of 4/5-4/9, and 32 students (49.25%) on the week of 4/12-4/16.

Figure 1. Percent of students from each campus and sports team screened per week offered.

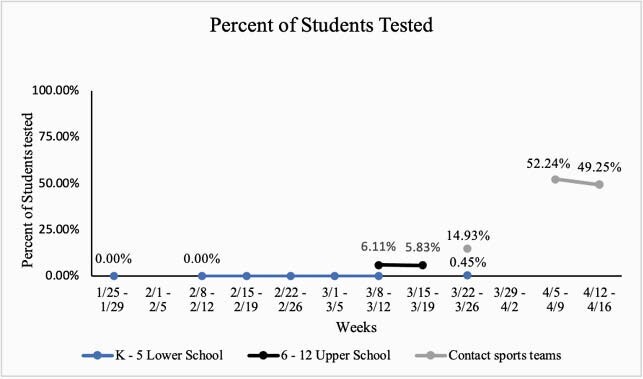

**Conclusion:**

Voluntary SARS-CoV-2 screening was not a feasible approach for detection of asymptomatically infected individuals due to low uptake, however in the same school, mandatory testing had high uptake and would be a feasible strategy.

**Disclosures:**

**All Authors**: No reported disclosures

